# Predicting prognosis and IDH mutation status for patients with lower-grade gliomas using whole slide images

**DOI:** 10.1038/s41598-021-95948-x

**Published:** 2021-08-19

**Authors:** Shuai Jiang, George J. Zanazzi, Saeed Hassanpour

**Affiliations:** 1 Department of Biomedical Data Science, Geisel School of Medicine at Dartmouth, Hanover, NH 03755 USA; 2 Department of Pathology and Laboratory Medicine, Dartmouth-Hitchcock Medical Center, Lebanon, NH 03756 USA; 3 Department of Computer Science, Dartmouth College, Hanover, NH 03755 USA; 4 Department of Epidemiology, Geisel School of Medicine at Dartmouth, Hanover, NH 03755 USA

**Keywords:** Prognostic markers, CNS cancer, Genetic markers, Machine learning

## Abstract

We developed end-to-end deep learning models using whole slide images of adults diagnosed with diffusely infiltrating, World Health Organization (WHO) grade 2 gliomas to predict prognosis and the mutation status of a somatic biomarker, isocitrate dehydrogenase (IDH) 1/2. The models, which utilize ResNet-18 as a backbone, were developed and validated on 296 patients from The Cancer Genome Atlas (TCGA) database. To account for the small sample size, repeated random train/test splits were performed for hyperparameter tuning, and the out-of-sample predictions were pooled for evaluation. Our models achieved a concordance- (C-) index of 0.715 (95% CI: 0.569, 0.830) for predicting prognosis and an area under the curve (AUC) of 0.667 (0.532, 0.784) for predicting IDH mutations. When combined with additional clinical information, the performance metrics increased to 0.784 (95% CI: 0.655, 0.880) and 0.739 (95% CI: 0.613, 0.856), respectively. When evaluated on the WHO grade 3 gliomas from the TCGA dataset, which were not used for training, our models predicted survival with a C-index of 0.654 (95% CI: 0.537, 0.768) and IDH mutations with an AUC of 0.814 (95% CI: 0.721, 0.897). If validated in a prospective study, our method could potentially assist clinicians in managing and treating patients with diffusely infiltrating gliomas.

## Introduction

Diffuse gliomas are the most common primary brain tumors in adults, and one of the most common causes of cancer death affecting young adults^1^. According to the World Health Organization (WHO) classification of tumors of the central nervous system^2^, the diffusely infiltrating gliomas are categorized into grade 1 to 4 based on histologic features such as mitotic activity, tumor cell pleomorphism, and the presence of necrosis and/or microvascular proliferation^2^. Lower-grade gliomas (LGG) refer to grade 2 and grade 3 gliomas, and the median survival time of patients with LGG is 7 years^3^. Predicting survival times for patients with LGG can inform treatment and promote shared-decision making between physicians and patients, and is of great interest in clinical practice. But this is a challenging task given the heterogeneity of this disease from histological, genetic, and clinical aspects, as well as the efforts required for obtaining the morphological and molecular features.

Prognostic factors for adult diffuse gliomas include age, gender, performance status, the extent of tumor resection, and intrinsic factors of the tumor including grade, isocitrate dehydrogenase (IDH) mutation, chromosome 1p/19q status, and MGMT promoter methylation^4,5^. Although tumor tissues are graded according to well-established histological criteria, this manual process is time-intensive and cannot provide detailed information for an accurate survival estimation. The development of deep learning models over the past few years provides unique opportunities to extract information from unstructured data such as whole slide images (WSIs)^6–8^. Several studies have used WSIs to predict the prognosis of patients diagnosed with diffuse gliomas and have shown promising results^9,10^. However, these models either were developed and evaluated for glioma patients with various grades (grade 2 to 4), or did not report the performance when only using WSIs. Information on the prognostic performance of WSI-based deep learning models for LGG is limited. A model trained using data across grade 2 to grade 4 cases can perform well in distinguishing high-risk patients from low-risk patients with different grades of gliomas; however, it might not be able to differentiate high-risk patients from low-risk patients within the same grade.

In addition to morphological features, IDH mutation is another important prognostic feature for glioma patients. IDH mutations are common in LGG patients with a prevalence of about 80%^11^, and are associated with more favorable outcome^3,12^ compared to IDH wild-type. Recent retrospective and clinical studies also suggest the presence of an IDH mutation is an important treatment indicator^9,13–19^, thus it is crucial to consider IDH mutation status in clinical treatment planning. However, investigating IDH mutational status can be time-consuming and expensive. If we can obtain the IDH mutation information directly from the histopathological slides, both time and cost could be significantly reduced. However, there has not been a study on using deep learning models to infer IDH mutation status based on WSIs for LGG patients, and it is not clear how the WSI-inferred IDH mutation status can affect the performance in predicting survival.

In this study, we set our focus on LGG and explored the use of deep learning models for survival and IDH mutation status predictions utilizing The Cancer Genome Atlas (TCGA) database. This is a more challenging task in comparison to previous studies due to the smaller sample size and less variation in patient outcomes. To overcome this challenge in our study, we used an ensemble deep learning framework and obtained pooled out-of-sample predictions from repeated random splits of the dataset to ensure the stability and quality of our results. This approach helps obtaining the distribution of the model performance on the entire dataset and makes the results not subject to unbalanced splitting. We additionally evaluated if the WSI-inferred IDH mutation status can be used for survival prediction when such information has not been directly measured for these patients.

## Results

### Model performance and comparison with clinical features for prognosis prediction

The average performance of the models with the chosen hyperparameters was 0.644 (standard deviation = 0.107) in the 32 separate test splits. The ensembled predictions achieved a C-index of 0.715 (95% CI: 56.9, 83.0) for the prognosis prediction task over the entire dataset (Table 1). Several demographic and clinical variables were considered for survival analysis in our study. A Cox-proportional hazards model of age achieved a C-index of 0.745. When our WSI risk scores were added to the Cox model, the C-index was improved to 0.765 (difference = 0.020, 95% CI: − 0.091, 0.100). Gender and race were unrelated to survival, with C-index close to 0.5. The C-index when using only a clinical variable (primary diagnosis) was 0.572. By adding our WSI-based risk scores, the C-index was increased to 0.689 (difference 0.117, 95% CI: − 0.003, 0.230) but still lower than WSI risk scores alone. IDH mutation status was another strong predictor with a C-index of 0.692 without WSI risk scores or 0.762 with WSI risk scores. When combining age and IDH mutations, the C-index was 0.774 (95% CI: 0.658, 0.863), and adding our WSI-based risk scores improved the C-index slightly to 0.784 (difference = 0.010, 95% CI: − 0.097, 0.085).

**Table 1:** Model performance statistics for survival prediction task and IDH mutation prediction task, evaluated among patients with grade 2 gliomas.

**Survival prediction performance: C-index [95% CI]**			
	Without WSI risk score	With WSI risk score	Difference
None	–	0.715 [0.569, 0.830]	–
Age	0.745 [0.627, 0.838]	0.765 [0.643, 0.865]	0.020 [− 0.091, 0.100]
Gender	0.509 [0.345, 0.630]	0.688 [0.528, 0.815]	0.179 [− 0.007, 0.352]
Race	0.520 [0.444, 0.554]	0.713 [0.568, 0.831]	0.193 [0.056, 0.322]*
Primary diagnosis	0.572 [0.437, 0.707]	0.689 [0.539, 0.822]	0.117 [0.003, 0.230]*
IDH mutations	0.692 [0.573, 0.807]	0.762 [0.602, 0.878]	0.070 [− 0.048, 0.161]
Age + IDH mutations	**0.774 [0.658, 0.863]**	**0.784 [0.655, 0.880]**	0.010 [− 0.097, 0.085]

**IDH mutation prediction performance: AUC [95% CI]**			
	Without WSI Predicted IDH Mutation Probability	With WSI Predicted IDH Mutation Probability	Difference
None	–	0.667 [0.532, 0.784]	–
Age	0.689 [0.552, 0.816]	0.726 [0.599, 0.845]	0.037 [− 0.053, 0.097]
Gender	0.536 [0.430, 0.643]	0.650 [0.507, 0.769]	0.114 [− 0.078, 0.240]
Race	0.567 [0.480, 0.650]	0.687 [0.560, 0.800]	0.120 [− 0.002, 0.229]
Primary diagnosis	0.519 [0.389, 0.641]	0.637 [0.472, 0.755]	0.118 [− 0.090, 0.261]
Age + Race	**0.711 [0.585, 0.834]**	**0.739 [0.613, 0.856]**	0.028 [− 0.051, 0.078]

95% confidence intervals were derived from 10,000 bootstrapping replications. **Bold** texts indicate the best performance for each column. * Indicates statistically significant difference (*p* < 0.05).

**Figure 1:**
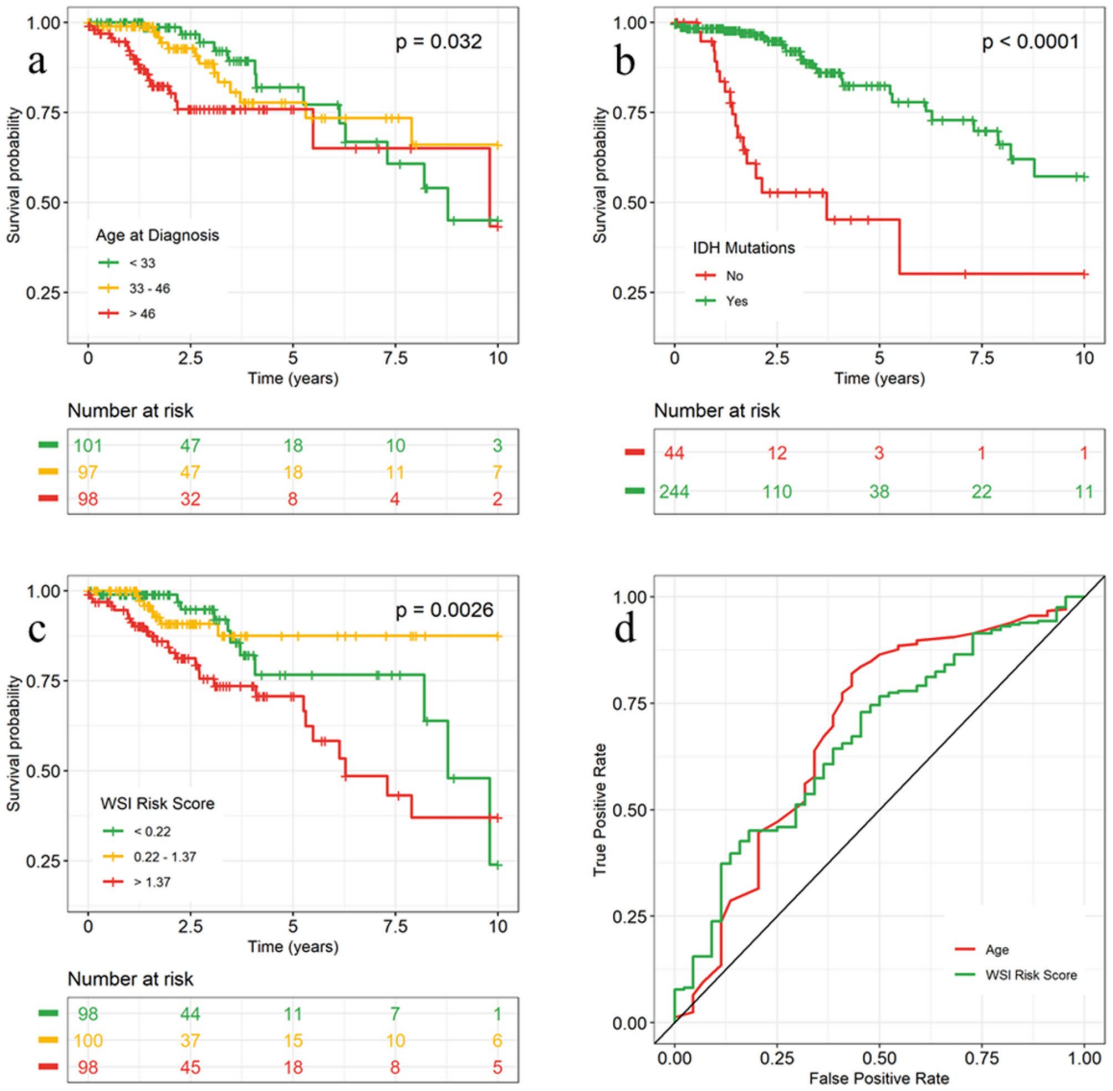
Kaplan–Meier curves and ROC curves were evaluated among patients with grade 2 gliomas. (**a**), (**b**), (b) Kaplan–Meier curves by age, IDH mutations, and WSI risk score, respectively. *P*-value was calculated by log-rank test. Age and WSI risk score were categorized in tertiles. (**d**) ROC curve for IDH mutation prediction.

We then partitioned age and WSI risk score into 3 categories with an equal number of patients (i.e., tertiles) to visualize the survival curve for each category. Figure 1a and c show both age and WSI risk scores can successfully identify high-risk patients (age > 46 years or WSI risk score > 1.37) shortly after diagnosis. However, patients with intermediate-risk were not significantly different from patients with low risk. The survival curve for patients with IDH mutations separated from the survival curve for IDH wild-type patients about half-year after diagnosis (Fig. 1b). Log-rank tests were significant for all three predictors.

In the WHO grade 3 cases (Supplementary Table S3), the WSI-based risk scores had lower performance alone (0.654, 95% CI: 0.537, 0.768). Age and IDH mutations together achieved a C-index of 0.786 (95% CI: 0.683, 0.877). When combining WSI risk scores, the model performance improved slightly to 0.792 (95% CI: 0.701, 0.876). Kaplan–Meier curves for patients with WHO grade 3 gliomas are shown in Supplementary Figure S1.

### Model performance and comparison with clinical features for IDH prediction

The AUC of the WSI-based models for predicting IDH mutations was 0.667 (95% CI: 0.532, 0.784) (Table 1 and Fig. 1d). In addition, age is a strong predictor of IDH mutations with an AUC of 0.689 (95% CI: 0.552, 0.816). Race is a weak predictor with an AUC of 0.567 (95% CI: 0.480, 0.650). Combining race and WSI-based scores, the AUC was increased to 0.687. When combining age and race, the AUC was 0.711 (95% CI: 0.585, 0.834). Including WSI-based scores raised the AUC to 0.739, with 0.028 improvement (95% CI: − 0.051, 0.078).

For WHO grade 3 cases (Supplementary Table S3 and Figure S1), the WSI-based scores can predict IDH mutations with an AUC of 0.814 (95% CI: 0.721, 0.897), which is much higher than the demographic and clinical predictors. When combining age, the AUC was 0.845 (95% CI: 0.759, 0.919), which is a statistically significant improvement over using only age as the predictor (0.122, 95% CI: 0.001, 0.198).

### Prognosis prediction using WSI predicted IDH mutation probability

Additionally, we explored if WSI-predicted IDH mutation probability can be used to replace IDH mutation status measurement in predicting the prognosis (Table 2 and Supplementary Table S4). Among patients with grade 2 gliomas, we found predicted IDH mutation probability alone achieved a C-index of 0.727, which is notably greater than the WSI risk score (0.715) and IDH mutations (0.692). When combining with age, the C-index increased to 0.767 (95% CI: 0.646, 0.862), but not as good as combining age and IDH mutations (C-index = 0.774). Finally, when combining predicted IDH mutation probability with age and WSI risk score, the C-index was 0.771, which was better than age and survival risk score (0.766), but not as good as combining age, survival risk score, and IDH mutations (0.784). Among patients with grade 3 gliomas, similarly, we found that the WSI-inferred IDH mutation probability could improve the performance of survival prediction. For example, combining WSI-inferred IDH mutation probability with age and WSI risk score could achieve a C-index of 0.771. Although this performance is lower than using IDH mutations measurement directly (C-index = 0.792), it is 0.014 higher than using age and WSI risk score alone (C-index = 0.757).

**Table 2:** Performance of survival prediction using predicted IDH mutation probability evaluated among patients with grade 2 gliomas.

	Without WSI predicted IDH mutation probability	With WSI predicted IDH mutation probability	Difference
None	–	0.727 [0.593, 0.834]	–
Age	0.746 [0.625, 0.838]	0.767 [0.646, 0.862]	0.021 [− 0.069, 0.091]
Gender	0.509 [0.344, 0.629]	0.704 [0.561, 0.823]	0.195 [0.041, 0.367]*
Race	0.520 [0.445, 0.554]	0.719 [0.585, 0.831]	0.199 [0.075, 0.322]*
Primary diagnosis	0.573 [0.433, 0.707]	0.700 [0.554, 0.818]	0.127 [0.001, 0.236]*
WSI risk score	0.715 [0.573, 0.831]	0.723 [0.574, 0.839]	0.008 [− 0.114, 0.081]
Age + WSI risk score	**0.766 [0.646, 0.866]**	**0.771 [0.647, 0.867]**	0.005 [− 0.083, 0.062]

95% confidence intervals were derived from 10,000 bootstrapping replications. **Bold** texts indicate the best performance for each column. * Indicates statistically significant difference (*p* < 0.05).

### Visualization of model predictions

**Figure 2:**
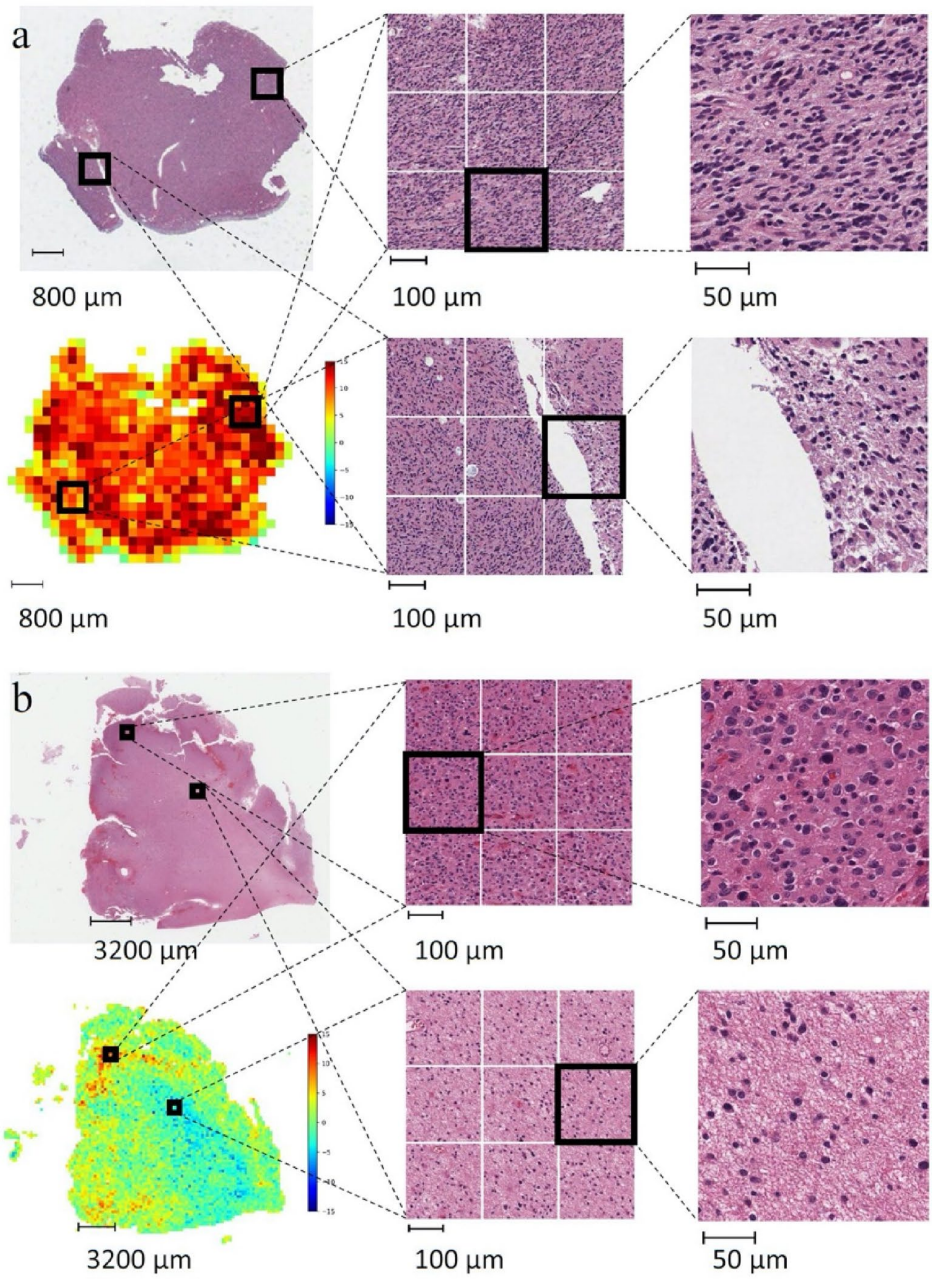
Example predictions on whole slide images for prognosis prediction. (**a**) A 59-year-old female patient diagnosed with mixed glioma, died 1.4 years after diagnosis. The predicted risk score is 10.77. (**b**) A 32-year-old man diagnosed with mixed glioma, died 6 years later. The predicted risk score is 0.65.

The average WSI-based risk scores across patients were 0.947 (standard deviation: 1.587). Prediction results on the whole slide and patch level are shown in Fig. 2 and Supplementary Figure S2. Increased tumor cell density and tumor cell atypia, i.e., increased nuclear size, hyperchromasia, and irregular nuclear contours, are associated with higher grade and worse prognosis. The images of the resection specimen in Fig. 2a show a diffusely infiltrating glial neoplasm with many areas of high cellularity and pleomorphism. This tumor was diagnosed at the time as an oligoastrocytoma (mixed glioma), and the patient died 1.4 years after diagnosis. The predicted risk score was high (10.77). The histology of this tumor differs dramatically from the one shown in Fig. 2b, which reveals only small foci of hypercellularity and atypia (insets, left). Much of the resection specimen from this 32-year-old man diagnosed with mixed glioma showed reactive astrogliosis and mildly infiltrated brain parenchyma (insets, right). The model’s low predicted risk score of 0.65 is consistent with the low-grade histologic features of this tumor. The patient’s relatively long survival of six years corroborated the model’s performance.

## Discussion

In this study, we have shown that by using deep learning models on WSIs, we are able to achieve promising results for predicting prognosis and IDH mutational status on the LGG dataset from the TCGA database. The performance of the deep learning model based on WSIs alone is better than the model based on the primary diagnosis and some demographic variables such as race and gender, but not as good as age at diagnosis. Combining WSI-based deep learning predictions with demographic and clinical features could further improve the model performance up to 0.784 to predict prognosis and 0.739 to predict IDH mutational status. We also found if WSI predicted IDH mutation probability is used instead of IDH mutation status measurement, we could still obtain a C-index of 0.771. Our results were further validated using the WHO grade 3 subset which was not used during the training and hyperparameter selection.

Age is the single best predictor for almost all the tasks evaluated, except for IDH mutation prediction among grade 3 glioma patients. However, gender, race, and primary diagnosis provided little value in the prognosis prediction. It has long been known that histopathological diagnosis of lower-grade gliomas does not adequately predict clinical outcomes due to interobserver variability^20^. And previous studies have identified age as an important prognostic factor using non-TCGA datasets, with older age associated with worse outcomes^21,22^. This could be due to the natural progression of the disease or that age is a proxy variable for many factors that could affect the survival of LGG patients, such as comorbidities. Similar to previous studies, we also found that IDH mutation is an important predictor of survival. Despite the strong prognostic value of age and IDH mutations, we still achieved a small yet consistent improvement in C-index when including WSI-derived predictions, demonstrating that our deep learning approach could extract complementary prognostic information from WSIs for developing a more accurate survival prediction framework.

Previous work on the application of deep learning models to LGG datasets is relatively limited. Studies using less restrictive data inclusion/exclusion criteria reported higher performance in the survival prediction task and IDH mutation prediction task. Specifically, Mobadersany et al.^9^ predicted survival of patients diagnosed with grade 2 to 4 gliomas from the TCGA database, and they obtained a C-index of 0.741 in the testing phase. The C-index of 0.715 achieved by the WSI risk scores in our study with only grade 2 patients is considerably lower likely due to the smaller sample size of our study population and less variation in the disease severity.

Of note, for the IDH mutation prediction task, Momeni et al. applied deep recurrent attention models using the TCGA dataset and obtained an AUC of 0.86^23^. In another study, Liu et al. achieved an AUC of 0.920 with a dataset combining 200 TCGA grade 2 to 4 cases and 66 private cases^24^. In contrast, the AUC of our model evaluated on the restrictive grade 2 dataset was only 0.667, possibly because most (85%) of the grade 2 LGG patients had IDH mutations. Although we explored both oversampling of the minority class and down-weighting the loss for the majority class, the performance could not be further improved on such an imbalanced dataset. However, on grade 3 dataset, which is more balanced in terms of IDH mutation status (68.6%), we observed a much higher AUC (0.814) using the same model. This indicates that our model does not lack the ability to distinguish IDH mutation status, but that the imbalanced dataset makes the objective evaluation challenging.

Additionally, we found that using the inferred IDH mutation probability estimated from the WSIs could help prognosis prediction. Among grade 2 patients, there was a 0.005 increase in AUC when including the inferred IDH mutation probability in addition to age and WSI risk score. Among grade 3 patients, such improvement was 0.014. While the prognosis prediction performance using IDH mutation probability was not as good as using IDH mutation measurement directly, it can still provide the LGG patients more accurate survival estimate when their IDH mutation status is not available. Determining IDH mutation status can be expensive and time-consuming, because only a small proportion of mutations can be currently identified by sequencing^25^. Our deep learning model can serve as a readily available tool for predicting IDH mutation status from WSIs without extra cost and waiting time.

During initial experiments, we noticed that for a single data split, the higher performance in the validation dataset does not necessarily translate to higher performance in the test dataset. This could be due to unbalanced sampling when the sample size is small. We also found that a discrepancy in validation and test AUC occurred in Liu et al.’s study^24^. For example, the AUC for their baseline model achieved an AUC of 0.920 on the test set, while in the validation dataset the AUC is 0.823. This highlights the difficulty in obtaining a balanced train/validation/test splitting with a limited sample size. Our adoption of the repeated data splits and pooling method can alleviate this problem.

There are several limitations to this study. First, the sample size in the study is relatively small and the number of lost to follow-up is substantial. With only 296 patients (among which 49 were observed at the endpoint and 44 were IDH wild-type), developing a deep learning framework is challenging. The small sample size also limited the power to detect statistically significant improvement using the predictions based on WSIs over only demographic and clinical information. Secondly, we did not evaluate the performance of our models on additional datasets; thus, the generalizability of this method needs further validation. Thirdly, only the IDH mutation status was considered as a molecular biomarker in this study. Other molecular biomarkers that are also important for LGG prognosis prediction, such as 1p/19q co-deletion, that were not included in the current study will be explored in future work. Lastly, the cause of death was not recorded in the TCGA dataset, thus our ground truth might not be accurate for all the samples which could affect the model performance.

Notably, histological information could only explain part of the variance in survival time. Other information, such as the location of the tumor, treatment, and comorbidities are also important determinants of the progression of the disease. In this study, we did not include important clinical data, such as treatment, in our analysis, as the detailed information was not available in the TCGA dataset. We will pursue expanding our dataset and include this additional relevant information in our analysis in future work. We expect incorporating additional demographic, clinical, and genetic/molecular information in our method could potentially further improve the ability to predict the prognosis of patients diagnosed with LGG.

## Materials and methods

### Data source

The digitized hematoxylin and eosin (H&E) stained whole slides used in this project were obtained from the TCGA database. TCGA database is de-identified and is publicly available on the Web. Therefore, this project does not meet the requirements of human subject research. Only grade 2 diffuse glioma patients were included for model development (number of patients = 307). There are two different types of whole slide images in this dataset, namely formalin-fixed paraffin-embedded (FFPE) slides and frozen section slides^26^. Since the frozen section slides contain many artifacts, we only included FFPE slides in our dataset (number of patients = 296, number of WSIs = 524).

Demographics and clinical information were also downloaded from the TCGA website. For the deceased patient, the follow-up time was derived from “days to death”. For patients who were alive at the last follow-up, the follow-up time was derived from “days to last follow-up”. IDH mutation status was derived from IDH1 and IDH2 mutation variables. Eight participants without IDH mutation information were excluded from IDH related analysis. Demographic and clinical information including age, gender, race, and primary diagnosis, were used in our analysis for comparison purposes. The average age of the patients in our dataset was 40.9 years with a standard deviation of 13.0 years. Among those, 55.7% were men, and the majority (91.6%) of the patients were white. The proportions of patients diagnosed as astrocytoma, oligoastrocytoma, and oligodendroglioma were 19.9%, 43.9% and 36.1%, respectively. 80.2% of the patients had an IDH1 mutation, while 4.5% had an IDH2 mutation (Supplementary Table S1).

To further evaluate the performance of our method, we obtained the grade 3 glioma cases from the TCGA database for testing purposes only (number of patients = 194, number of whole slides images = 319). The data processing procedure for grade 3 cases is the same as the grade 2 cases. The distribution of demographic variables was similar to grade 2 cases, and IDH mutations were present in 68.6% of the patients (Supplementary Table S2).

### Preprocessing of whole slide images

As WSIs are large and cannot fit in GPU memory, several preprocessing steps were taken to extract patches from the original images. We loaded the WSIs at the magnification factor of 10 × (1 μm/pixel) and extracted patches with a size of 224 × 224 pixels without overlap. Background patches were excluded by using color thresholding. A total of 1,887,767 patches were generated through this process.

### Model architecture

**Figure 3:**
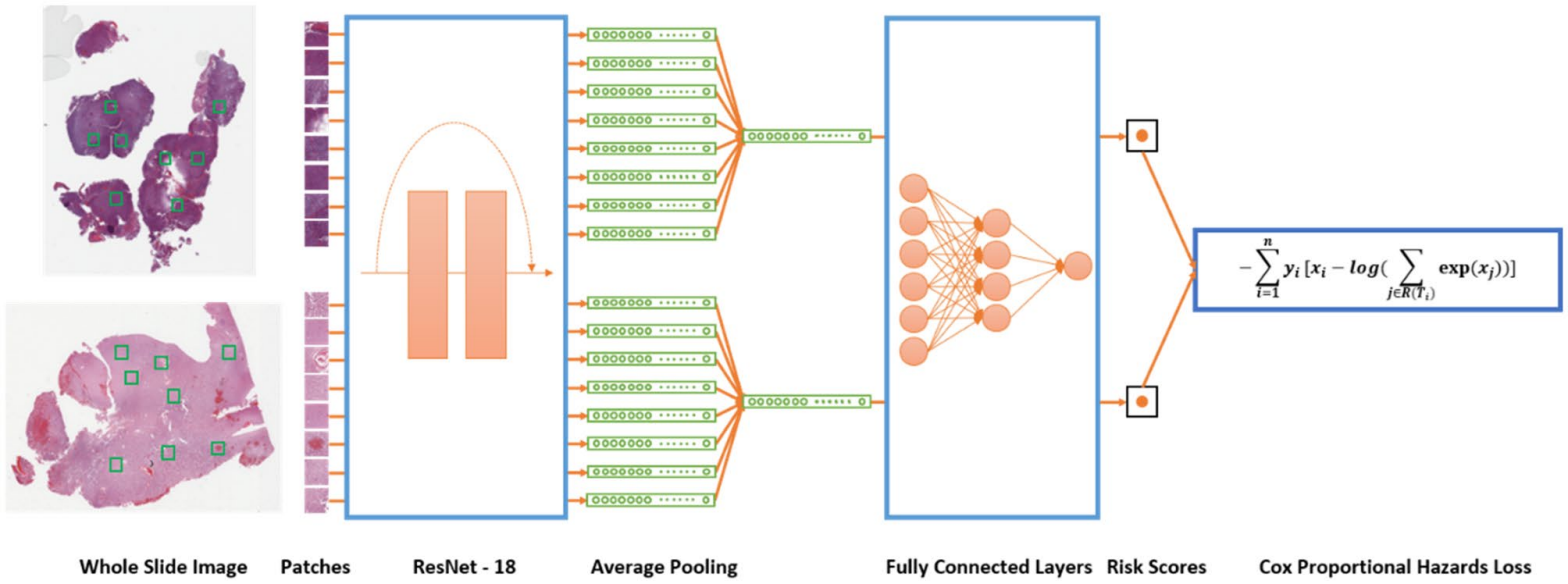
An overview of the deep learning pipeline for prognosis prediction. Patches of size 224 × 224 × 3 are randomly sampled from whole slide images at a 10 × magnification level. The ResNet-18 Convolutional Neural Network transformed each patch into a 512 × 1 vector. Average pooling is performed at the patient level. The patient level vectors then go through a two-layer fully connected network with a final output size of 1, which can be interpreted as risk scores. Cox proportional hazards loss is calculated using the risk scores with consideration of follow-up time and vital status. The gradient is calculated and backpropagated through the fully connected layers and the ResNet-18 layers to train the entire model.

For the prognosis prediction task, our model architecture is adapted from the proposed work by Wulczyn et al.^27^ and is illustrated in Fig. 3. In summary, for each batch, $n$ participants were randomly chosen from the training dataset. For each participant, $k$ patches were randomly selected. These patches were fed into a deep learning model. The ResNet-18 model with pre-trained ImageNet weights was used as the backbone model^28^, and a fully-connected layer was replaced by an identity layer. The output size for each patch was 512. We then averaged the feature vectors over $k$ patches for each participant and used the pooled features for risk estimation through a subsequent two-layer neural network with 128 neurons and 1 neuron for each layer, respectively. The final output can be interpreted as risk scores and the loss is calculated as the negative log Cox partial likelihood, which is defined as

$$J\left(\beta \right)= -\frac{1}{n}{\Sigma }_{i=1}^{n}{y}_{i}[{x}_{i}-\mathrm{log}({\Sigma }_{\mathrm{j}\in \mathrm{R}\left({\mathrm{T}}_{\mathrm{i}}\right)}\mathrm{exp}({x}_{j}))]$$

where $n$ is the number of patients, ${x}_{i}$ is the risk score, ${y}_{i}$ is the event indicator (0 for alive and 1 for death), $R({T}_{i})$ is the risk set at the event time of $i$th patient.

The model architecture for the binary IDH mutation prediction task is similar to the one for prognosis prediction, except that the final output size is 2. Since the percentage of participants with IDH mutations was much larger than that of participants without IDH mutations, we used weighted cross-entropy loss to handle the imbalanced dataset by assigning a larger weight to cases without IDH mutations.

During validation, 100 random patches were selected for each patient in the validation group for a balance between variations and efficiency. All the patches were used when making out-of-sample predictions for cases in the test set.

### Model evaluation metrics

Concordance index (C-index), which is defined as the proportion of concordant pairs among all possible pairs, was used as the evaluation metric of our prognosis prediction model. Area under ROC (receiver operating characteristic) curve (AUC) was used as the evaluation metric for the binary classification tasks.

### Training-validation data splits

The data splitting was performed at the patient level to avoid the information leak across partitions. Due to limited training data, to ensure more balanced group splits, we first sorted the patients by vital status and follow-up time, then created multiple 4-patient-blocks. Within each block, we assigned 2 patients to the training group and 1 patient to each of the validation and test groups. This random splitting was repeated 8 times for hyperparameter tuning, and was repeated another 24 times for model evaluation (as explained below).

### Hyperparameter tuning

Within each random data split, our deep learning model was fit on the training split, with its performance monitored using the validation split. When the training is finished, an out-of-sample prediction was obtained for the test dataset. We repeated this process in all of these 8 repetitions, and used the average validation performance metrics to choose the best set of hyperparameters. The final set of hyperparameters chosen was batch-size of $n=8$ patients with $k=8$ patches for each individual (64 patches per batch in total), an initial learning rate of 1e−4 for the fully connected layers and 3e−7 for the convolutional layers.

Data augmentation methods, such as random horizontal and vertical flips and color jittering, were used during training time. To mitigate overfitting, we applied an L1 penalty with a regularization strength of 0.01 on the fully connected layers. Adam optimization was used for training. Cosine annealing was used as the learning rate scheduler. Each model was evaluated when every 20,480 (i.e., 320 steps) patches were used and training was stopped after 96 thousand steps.

### Bootstrapping on out-of-sample predictions

After training and hyperparameter tuning across the first 8 random splits, we trained the models with the same hyperparameters using the additional 24 random splits. These 32 models provided 32 out-of-sample predictions. The test set size for each model was one-fourth of the total dataset. Because each participant was selected into the test dataset with a probability of 0.25, the number of out-of-sample predictions for a participant follows the Poisson distribution with a mean of 8 (min = 2, max = 18). We ensembled all the out-of-sample predictions by averaging them as the final prediction.

Subsequently, we performed a bootstrapping method to evaluate the model performance. To do so, we randomly selected 296 observations from the entire dataset with replacement as the training dataset (about 63% of the patients). A statistical model (Cox or logistic) using demographic and clinical information with/without deep learning predictions was fit on the training dataset. The participants who were not selected formed the test dataset (about 37% of the patients) and were used to evaluate the performance of the statistical model. We repeated this process 10,000 times to estimate the distribution of C-index and AUC without or with deep learning predictors as well as their difference. The deep learning framework was implemented in PyTorch (version 1.1.0). The statistical tests were performed using R (version 3.6.1).

### Results visualization

Kaplan–Meier curves were used to present the observed survival probability over time by tertiles (i.e., 33rd and 67th percentiles) of age and WSI risk score, and IDH mutation status. The Kaplan–Meier curves were replicated for patients with grade 3 gliomas using the same cut-offs. For the IDH mutation prediction task, ROC curves were plotted with age and WSI-based IDH mutation probability as the predictor.

To visualize the model performance at the whole slide level, we selected two patients from the prognosis prediction task and another two from the IDH mutation prediction task. For the prognosis prediction task, we chose one patient who died shortly after diagnosis, and another patient who survived at least 5 years after diagnosis. For the IDH mutation prediction task, we chose one patient with an IDH mutation and one without. One whole slide image was selected for each patient. Representative regions from the slide were chosen for a more detailed view.

## Supplementary Information

Supplementary Information.

## Data Availability

This project's source of data is the TCGA database, which is publicly available on the Web (https://portal.gdc.cancer.gov/projects/TCGA-LGG).

## Code availability

This project's source code is publicly available at https://github.com/BMIRDS/WSI-PLP.
